# A Novel Role for Lupeol in Hepatocellular Carcinoma Treatment via Promoting Autophagy to Suppress Exosome Secretion

**DOI:** 10.1002/fsn3.71654

**Published:** 2026-03-19

**Authors:** Xin Zhang, Kehan Chen, Chuyao Zheng, Dai‐yuan Liao, Jian‐Song Wang, Ling‐Li Wang

**Affiliations:** ^1^ School of Pharmaceutical Sciences Guangzhou University of Chinese Medicine Guangzhou People's Republic of China; ^2^ School of Life and Health Technology Dongguan University of Technology Dongguan People's Republic of China

**Keywords:** autophagy, exosomes, Lupeol, malignant hepatocellular carcinomas

## Abstract

Hepatocellular carcinoma (HCC) is a highly lethal malignancy presenting a considerable challenge due to limited therapeutic options. This study investigates the anti‐cancer mechanisms of Lupeol, a natural triterpenoid. Our results demonstrated that Lupeol exhibited significant anti‐proliferative effects on HepG2 cells with an IC_50_ of 42.82 μM at 24 h. Furthermore, Lupeol treatment significantly inhibited cell migration and suppressed the secretion of exosomes, as evidenced by the downregulation of exosome markers (CD63, TSG101) (*p* < 0.05). Mechanistic analysis revealed that Lupeol induces autophagy, marked by the upregulation of LC3‐II and Beclin‐1. Importantly, blocking autophagy with the inhibitor 3‐MA prevented the Lupeol‐induced reduction of CD63 and restored exosome secretion (*p* < 0.05). These findings suggest that Lupeol inhibits HCC progression by inducing autophagy to suppress exosome secretion, rather than merely acting as a correlative factor. This study elucidates a novel mechanism where Lupeol redirects multivesicular bodies toward the autophagic‐lysosomal pathway, highlighting its potential as an effective therapeutic strategy for HCC.

## Graphical abstract

Schematic representation of the mechanism by which Lupeol inhibits HCC progression via the autophagy‐exosome axis.

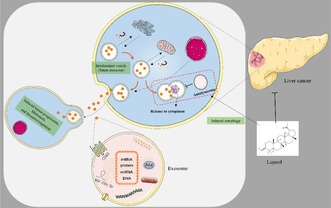

## 1 Introduction

Primary liver cancer, specifically hepatocellular carcinoma (HCC), poses a significant challenge to global health (Llovet et al. 2021). HCC is the predominant form of liver cancer, accounting for approximately 70% of cases worldwide. Moreover, it ranks among the seven most prevalent cancers globally and holds the second position in terms of cancer‐related mortality rates. Available evidence indicates that HCC is characterized by a bleak prognosis, exhibiting high incidence and mortality rates on a global scale. Liver cancer presents numerous treatment options, with liver transplantation or surgical resection being frequently employed for curative purposes (Anwanwan et al. 2020). The ailment exhibits a considerable recurrence rate, thereby imposing a significant burden on patients and their families. Consequently, it becomes imperative to explore potential therapeutic mechanisms for the treatment of HCC (El Dika et al. 2021).

Exosomes, which are extracellular vesicles with a diameter of 30–100 nm, are widely distributed in bodily fluids (Kalluri and LeBleu 2020). Exosomes play diverse roles in various diseases, including cancer. In HCC, the composition of RNAs and proteins in exosomes derived from HCC differs from those derived from normal hepatocytes (Zhang and Yu 2019). Exosomes from HCC could remodel the tumor immune environment through various mechanisms, thereby modulating anti‐HCC immune responses. It has been reported that exosomes derived from HepG2 cells can activate the MAPK (Ras–Raf–MEK–ERK) signaling pathway, which inhibits apoptosis by preventing caspase cleavage (Sasaki et al. 2019). It was found that exosomal miR‐30a played a significant role in the suppression of autophagy (Kok and Yu 2020). tumor‐derived exosomes have been shown to have crucial roles in various stages of the invasion and metastasis process, such as angiogenesis, epithelial‐mesenchymal transition (EMT), invasion, migration, and the formation of a premetastatic niche (Ge et al. 2020). Hence, the utilization of exosome‐based therapy presents a potentially innovative strategy for addressing HCC, with the potential for enhancing exosome therapeutics through the identification of factors that augment exosome secretion.

Autophagy, a cellular process involving the delivery of cellular components to lysosomes, has been found to inhibit the invasion and migration in tumor development (Li et al. 2020). The relationship between autophagy and exosomes is reported to be competitive (Raudenska et al. 2021), as both processes rely on the formation of multivesicular bodies (MVBs) (Buratta et al. 2020). MVBs are essential for autophagy, contributing to the formation of autolysosomes, while exosomes also require MVBs for their release (Guo et al. 2026; Jeppesen et al. 2019). Therefore, it is hypothesized that up‐regulating autophagy levels may lead to a down‐regulation of exosome levels in HCC.

Lupeol is a natural pentacyclic triterpenoid, which is abundantly present in diverse plant and fruit (de Seabra Rodrigues Dias et al. 2021; Zhu et al. 2018). Ongoing research endeavors have unveiled the molecular mechanisms underlying the effects of lupeol, primarily its anti‐tumor properties (Liu et al. 2021). For instance, lupeol has been found to upregulate the expression of P53, which subsequently induces the expression of Bax protein and activates caspase‐3 (Bhattacharyya et al. 2017). This cascade of events ultimately leads to apoptosis of head and neck cancer cells. Additionally, lupeol inhibits the invasion and metastasis of MDA‐MB‐231 cells through the PI3K/AKT/NF‐κB pathway (Li et al. 2022). Significantly, our prior investigation also revealed the capacity of lupeol to stimulate autophagy, thereby ultimately mitigating the progression of triple negative breast cancer, its proliferation, and metastasis (Zhang, Gao, et al. 2022). Considering the significant interconnection observed between autophagy, exosomes, and tumor development, it is plausible to suggest that lupeol may exert a pivotal influence on the process of exosome secretion and autophagy. Consequently, this phenomenon could potentially be harnessed as a viable therapeutic approach for the treatment of hepatic cancer.

Building upon prior research, our objective was to identify the potential mechanisms and therapeutic targets of lupeol against HCC, thereby establishing a solid groundwork for the development of lupeol‐based drugs and their subsequent clinical application.

## 2 Methods

### 2.1 Cell Culture and Compounds

The HepG2 cells were purchased from the Cell Bank of the Chinese Academy of Sciences (Shanghai, China) and were authenticated via STR profiling and confirmed negative for mycoplasma contamination. Cells were cultured in DMEM with high glucose (Gibco, USA) and 10% of FBS at 37°C in 5% CO_2_. Lupeol was bought by MedChemExpress (HY‐N0790); The CFDA‐SE kit (Exosomes fluorescent labeling) was bought from Good Laboratory Practice Bioscience (GLPBIO, America), 3‐MA (autophagy inhibitor) was acquired from Cell Science and Nature (CNSpharm, America), All compounds were dissolved in DMSO, and diluted with DMEM to the desired concentration with DMSO concentrations not exceeding 0.5%.

### 2.2 Cell Viability Assay and Colony Formation Assay

Each well of a 96‐well plate was seeded with HepG2 cells and LO_2_ cells (3000 cells per well) for the purpose of assessing the cytotoxicity of lupeol using the CCK8 assay. The cells were incubated in a 37°C/5% CO_2_ incubator for 24 and 48 h, and then treated with lupeol at concentrations of 0, 1.5625, 3.125, 6.25, 12.5, 25, 50, and 100 μM for 24 and 48 h, respectively. Prior to analysis, 10 μL of CCK8 reagent was added to each well and incubated for 4 h. The absorbance at 450 nm was measured using a microplate reader.

Each well of a six‐well plate was initially seeded with HepG2 cells (300–500 cells per well) and incubated with 37°C/5% CO_2_ for 24 h. Subsequently, the cells were treated with varying concentrations of lupeol and cultured for a period of 2 weeks, with media changed every 3 days. Following the incubation period, the cells were washed twice with PBS, fixed with paraformaldehyde for 30 min, and stained with a 0.1% solution of crystal violet (Solarbio, Beijing, China) for 30 min. The stained cells were then air‐dried overnight. The intensity of the crystal violet staining was quantified through Image J software (version 2.0.0).

### 2.3 Wound Healing Assay

HepG2 cells were cultured in six‐well plates and maintained in a controlled environment at 37°C with 5% CO_2_. A linear scratch was created when the cells reached approximately 90% confluence. Subsequently, the cells were exposed to different concentrations of lupeol for a duration of 24 h. The migratory behavior of the cells was captured through photographs at 0, 12, and 24 h, and the resulting data was analyzed using Image J software (version 2.0.0) for quantitative comparison.

### 2.4 Immunofluorescence (IF) Analysis

Exosomes were isolated from HCC using an exosome isolation kit (MEILUN BIO, MA0402‐T). To label the exosomes, CFSE (GLPBIO, America) was used. The exosome suspension was mixed with CFSE at a final concentration of 5 μM and incubated at 37°C for 30 min. The reaction was stopped by adding an equal volume of PBS containing 10% FBS. The labeled exosomes were then washed with PBS to remove any unbound CFSE and resuspended in PBS. The labeled exosomes were added to the cell culture medium of liver cancer cells at a predetermined concentration. The cells were incubated with the exosomes for 24 h to allow for internalization. After 24 h of incubation with labeled exosomes, lupeol was added to the cell culture medium at various concentrations (10, 20, 40 μM) for an additional 24 h. A control group without lupeol treatment was also included.

The level of cellular autophagy was examined using the Autophagy detection kit (Bestbio, China). Finally, the nucleus was counterstained with DAPI (G1012, Servicebio, China), and immunofluorescence was captured using a NIKON ECLIPSE C1 fluorescence microscope (Tokyo, Japan).

All results were acquired using a confocal laser scanning microscope (ZEISS), and the fluorescence intensity of CFSE‐labeled exosomes was quantified using image analysis software. The fluorescence intensity was normalized to the total cell number counted in each group.

### 2.5 Cell Treatment and Autophagy Inhibition Assay

HepG2 cells were seeded into six‐well plates at a density of 2 × 10^5^ cells per well and allowed to adhere overnight at 37°C. Upon reaching approximately 70%–80% confluence, the cells were randomly divided into control and treatment groups. For the autophagy inhibition experiments, cells were pre‐treated with 3‐Methyladenine (3‐MA) at a final concentration of 5 mM for 1 h to block autophagic flux. Subsequently, Lupeol (40 μM) was added to the culture medium, and the cells were co‐incubated for an additional 24 h. The control group received an equivalent volume of DMSO vehicle (final concentration < 0.1%). After treatment, cells and supernatants were collected for subsequent experiments.

### 2.6 Western Blot Analysis

The total protein content of cells and tumor tissues was extracted using RIPA lysate (P0013B, Beyotime, China) and quantified using the BCA kit. A total of 30 μg of protein per lane was separated by SDS‐PAGE and transferred onto PVDF membranes. Subsequently, the membranes were probed with various primary antibodies diluted at 1:1000 overnight at 4°C, including CD63 (RRID: AB‐2837603), TSG101 (RRID: AB‐2841675), HSP70 (RRID: AB‐2837950), LC3 (RRID: AB‐2844592), Beclin‐1 (RRID: AB‐2837614), P62 (RRID: AB‐2837869), ATG7 (RRID: AB2838097), N‐cadherin (RRID: AB2835344), E‐cadherin (RRID: AB‐2833315), and Vimentin (RRID: AB‐2835318). All antibodies were purchased from Affinity Biosciences (China). Proteins were incubated and subsequently subjected to detection with HRP‐conjugated secondary antibodies (1:5000) (RRID: AB‐2839429). The detection was performed using ECL advance reagent (Affinity Biosciences, LOT#1927B02, China) and visualized with a Bioworld ComplexTM2000 developer (Tokyo, Japan), while β‐actin (RRID: AB‐2839420), GAPDH (RRID: AB‐2839421) and tubulin beta (RRID: AB‐2827688) were employed as loading controls, and the gray value analysis was conducted using Image J software (version 2.0.0).

### 2.7 Real‐Time PCR

The extraction of total RNA from cells and tumor tissue was conducted using the RNA extraction kit (Haigene, China). Quantitative real‐time PCR was performed to analyze the mRNA expressions of LC3, Beclin‐1, P62, CD63, TSG101, HSP70, Vimentin, E2F2, and mir‐23a‐3p. The primer sequences used in the PCR are provided in Table 1. The relative expression levels of the respective mRNAs were determined using the $2^{-∆∆C_{t}}$ method with GAPDH serving as the endogenous reference.

**TABLE 1:** The primer sequences of genes.

Genes	Primer sequence (5′–3′)
Beclin‐1	F: ACCGAGTTCCTGCTGCCCTAC
	R: TGCCTTGGTCCACTGCTCCTC
P62	F: TGATTGAGTCCCTCTCCCAGATGC
	R: CCGCTCCGATGTCATAGTTCTTGG
LC3‐II	F: GTCAGCGTCTCCACACCAATCTC
	R: ACAATTTCATCCCGAACGTCTCCTG
Vimentin	F: CCTTCGTGAATACCAAGACCTGCTC
	R: AATCCTGCTCTCCTCGCCTTCC
CD63	F: TTCAACGAGAAGGCGATCCATAAGG
	R: TTCACGAGGCAGCAGGCAAAG
TSG101	F: CCTGCCACAACAAGTTCTCAGTACC
	R: TCCTCCTTCATCCGCCATCTCAG
HSP70	F: ACGCCAATGGTATCCTGAATGTGTC
	R: CAGCCTTGTACTTCTCTGCCTCTTG
E2F2	F: CCCGTCGTCCCTGAGTTCCC
	R: CCAGCGAAGTGTCATACCGAGTC
GAPDH	F: TGACATCAAGAAGGTGGTGAAGCAG
	R: GTGTCGCTGTTGAAGTCAGAGGAG
U6	F: GCTACAGGATGCGGCAAGGAAG
	R: AATGAAAGAGGGAGGGGAAGAGGAG
mir‐23a‐3p	F: GCGATCACATTGCCAGGG
	R: AGTGCAGGGTCCGAGGTATT

### 2.8 Mouse Xenograft Models

All animal experiments conducted in this study were granted approval by the Ethics Committee of Guangzhou University of Chinese Medicine, with the permit number ZYD‐1‐656, in Guangzhou, China. Male BALB/c nude mice were obtained from Guangzhou Vital River Laboratory Animal Technology Co. Ltd. All animal experiments comply with the ARRIVE 2.0 guidelines. To establish a subcutaneous xenograft tumor model, a total of 24 male BALB/c nude mice (4 weeks old) were subcutaneously injected with HepG2 cells (at a concentration of 1–2 × 10^6^ cells). Once the tumor size reached 100 mm^3^, which typically occurred approximately 5 days after injection, the mice were randomly divided into four groups with 6 mice per group. These groups include a control group treated with corn oil and three experimental groups receiving different doses of the treatment (20 mg/kg/2 days, 40 mg/kg/2 days, 80 mg/kg/2 days).

The experiment spanned a duration of 21 days, during which the tumor size and mouse weight were measured every 2 days and volume was calculated as *V* = (*L* × *W* ^2^)/2. Humane endpoints were defined as tumor volume exceeding 1500 mm^3^ (or 20 mm diameter), weight loss > 20%, ulceration, or severe distress. Animals were monitored daily, and no mice reached these endpoints or died prior to the study's conclusion. Upon completion of the experiment, all mice were euthanized through cervical dislocation. Subsequently, the tumors were extracted, weighed, and imaged. A portion of the tumor tissues was preserved in liquid nitrogen for western blot analysis, while another portion was fixed with formaldehyde for subsequent histopathological examination. We confirm that all animal methods were carried out in accordance with relevant guidelines and regulations.

### 2.9 Immunohistochemistry (IHC) Analysis

The tumor sections were immersed in an EDTA antigenic retrieval reagent (pH 8.0) and subjected to microwave‐assisted antigenic retrieval. Subsequently, the slides were incubated at 4°C with LC3 antibody (1:500) and CD63 antibody (1:500). A secondary antibody conjugated with HRP polymer was then applied for 50 min, followed by development with DBA advance reagent using a microscope.

### 2.10 Differentially Expressed Genes and Survival Analysis in TCGA Databases

The Cancer Genome Atlas (TCGA) (https://portal.gdc.cancer.gov/), a project supported by the National Cancer Institute (NCI) and National Human Genome Research Institute (NHGRI), has generated comprehensive, multi‐dimensional maps of the key genomic changes in various types of cancers. In order to obtain a consensus of differentially expressed genes, gene expression quantification data and clinical information of HCC patients were downloaded from the TCGA database (371 tumor samples vs. 276 normal samples). All data were used for variation analysis by wilcox‐tests method with the tool of R4.3.1. To see whether these 3 genes were related to prognostic significance, survival analysis was performed in the R environment. We used clinical information to plot the survival curves for 1/2 of patients with higher expression of a specific gene versus the 1/2 of patients with lower expression of this gene (*p* < 0.05).

### 2.11 Statistical Analysis

The data were presented as the mean ± standard deviation from three independent experiments (*n* = 3). Statistical analysis of the differences among the groups was performed using SPSS (version 25.0), employing a one‐way analysis of variance. Significance levels were denoted as follows: ^#^ *p* < 0.05; ^##^ *p* < 0.01; ^###^ *p* < 0.001.

## 3 Results

### 3.1 Lupeol Suppresses the Proliferation and Migration of HepG2 Cells In Vitro

**FIGURE 1:**
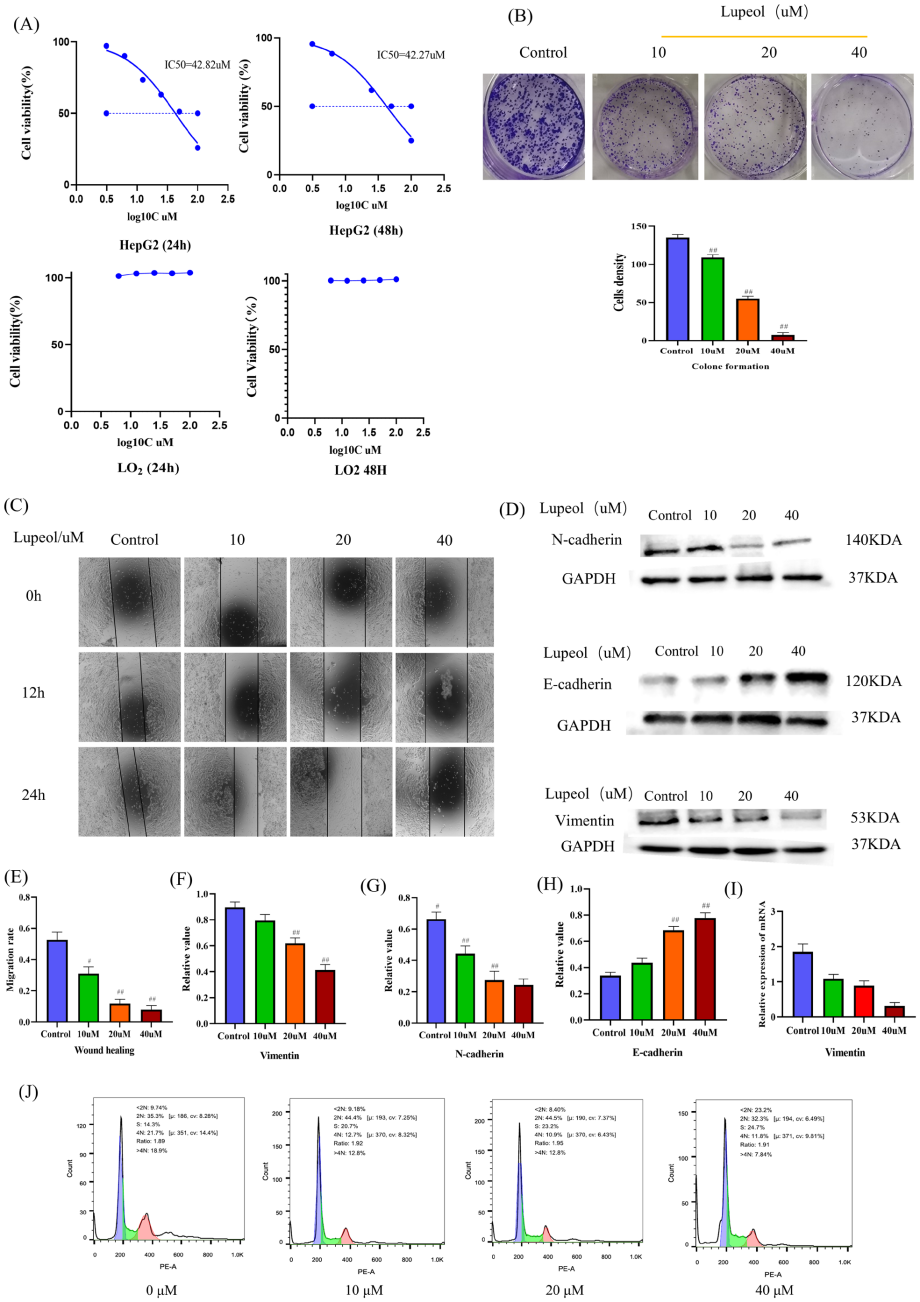
Lupeol inhibits HepG2 cell viability, migration, proliferation and blocks HepG2 cell cycle in S. (A) CCK8 assays were performed to assess cell viability and the results were analyzed by Prism 8.0.2; (B) colony formation assay results; (C, E) the results of cell migration via the scratch assay; (D, F–H) WB analysis of the levels of E‐cadherin, N‐cadherin, Vimentin in HepG2 cells treated with different concentrations of lupeol, with the results analyzed by Prism 8.0.2, ^#^ *p* < 0.05, ^##^ *p* < 0.01, compare to the control group. (I) RT‐PCR analysis of the mRNA expression of Vimentin, with the results analyzed by Prism 8.0.2, ^#^ *p* < 0.05, ^##^ *p* < 0.01, compare to the control group. (J) The results of cell cycle.

To investigate the potential anti‐proliferative effect and toxicity of lupeol extracted from the liver, cellular assessments were conducted using the CCK8 assay. HepG2 cells were treated with varying concentrations of lupeol to determine its impact. The results depicted in Figure 1A indicated that lupeol significantly inhibited the proliferation of HepG2 cells in a concentration‐dependent manner, with IC_50_ values of 42.82 and 42.27 μM at 24 and 48 h, respectively. Furthermore, lupeol did not exhibit any toxic effects on LO_2_ cells. To further confirm the anti‐proliferation effect of lupeol, a colony formation assay was also performed, and the lupeol concentrations of 20 and 40 μM were shown to be significantly inhibited compared with the control group in the colony formation assay (*p* < 0.05) (Figure 1B).

To assess the anti‐migration efficacy of lupeol in HepG2 cells, wound healing assays were employed, and the findings are depicted in Figure 1C,D. The results demonstrate that lupeol exhibits a substantial concentration‐dependent inhibition of the motility of HepG2 cells (*p* < 0.05). The Western blot assay (Figure 1E–H) demonstrated that the administration of lupeol resulted in the inhibition of N‐cadherin and Vimentin expression (*p* < 0.05), while the expression of E‐cadherin exhibited an opposite trend (*p* < 0.05). The mRNA expression levels of Vimentin were observed to be down‐regulated with increasing concentrations of lupeol (*p* < 0.05) (Figure 1I) It is well‐established that N‐cadherin, E‐cadherin, and Vimentin are proteins closely linked to EMT (Pastushenko and Blanpain 2019). These findings strongly indicate that lupeol effectively inhibits EMT in HepG2 cells. Additionally, flow cytometry was employed to examine the impact of lupeol on the cell cycle of HepG2 cells, which showed that the cell cycle was arrested in the *S* phase (Figure 1J) (*p* < 0.05).

### 3.2 Transcriptome Profiling of HCC Cells Treated With Lupeol

**FIGURE 2:**
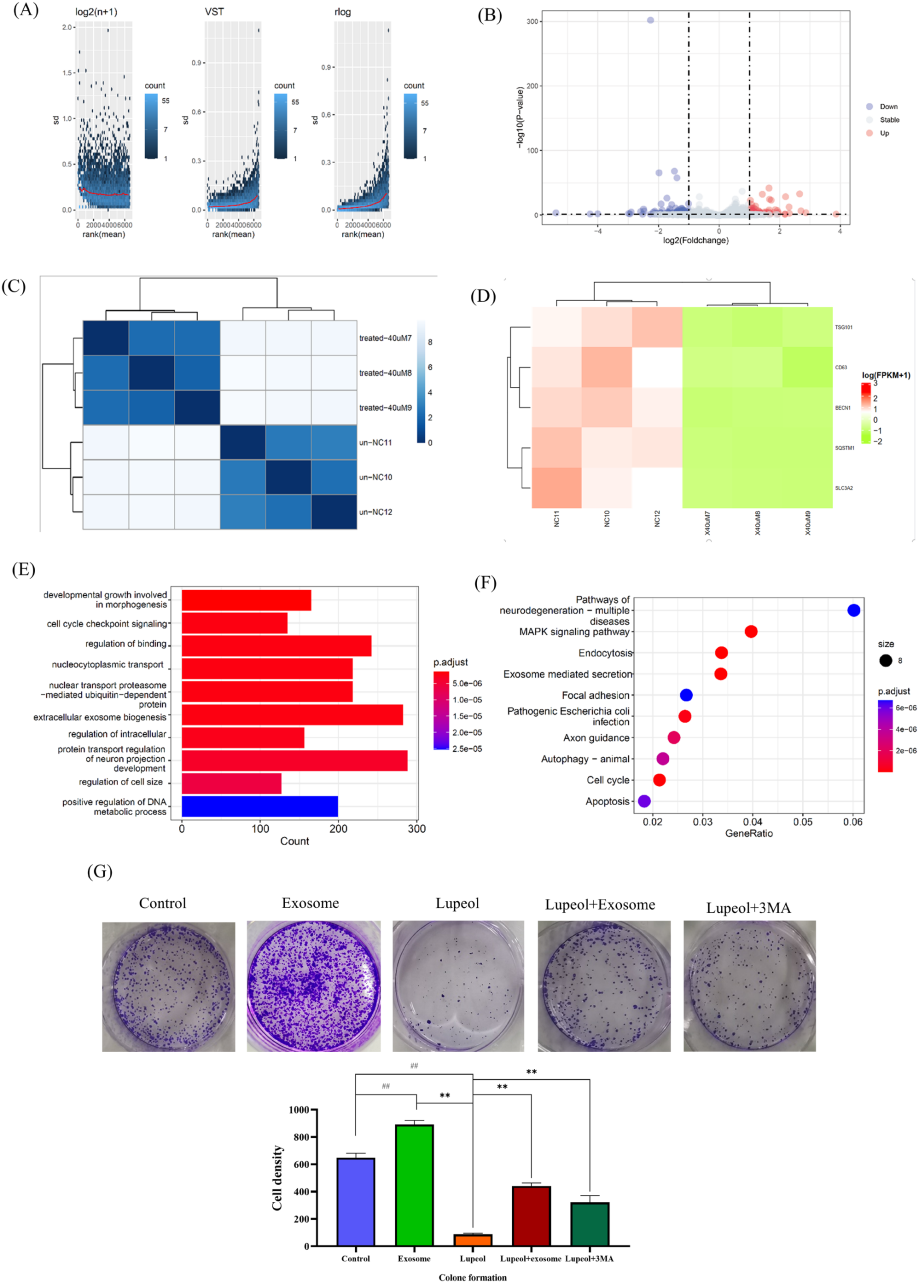
The transcript of HepG2 cells was analyzed before and after administration: (A) The data was standardized using log2 (*n* + 1), VST, and rlog methods. (B) Principle components analysis shows a total of 6624 genes, among these genes, 484 were found to be up‐regulated and 329 were down‐regulated. (C) Cluster thermography analysis revealed minimal differences in gene expression between the 40 μM group and the model group. (D) Gene thermography analysis was performed to further investigate these findings. (E) GO functional enrichment analysis. (F) KEGG pathway enrichment analysis. (G) Clone formation analysis, Lupeol (40 μM) effectively inhibited the proliferation of HepG2 cells (*p* < 0.05), in the Lupeol+Exosome group, exosomes were found to inhibit certain effect of Lupeol and promote the proliferation of HepG2 cells, with the results analyzed by prism 8.0.2, ^#^ *p* < 0.05, ^##^ *p* < 0.01, compared with the control group.

We next conducted a transcriptomic analysis using RNA‐seq. As illustrated in Figure 2A, the transcriptomic data was standardized using the log2 (*n* + 1), VST, and rlog methods, with the VST method yielding superior results. This standardized VST data was then used to perform a gene expression difference analysis (Figure 2B), which identified a total of 6624 genes. Among these, 484 genes were up‐regulated and 329 genes were down‐regulated. Cluster thermography analysis revealed minimal differences in gene expression between the 40 μM group and the model group, but significant differences were observed between these two groups (Figure 2C). Gene thermography analysis was further conducted to corroborate these findings. As a result, TSG101, CD63, BECN1, SQSTM1, and SLC3A2 were found to be the most significantly different between the 40 μM and NC groups, with TSG101 and CD63 being the marker genes of exosomes (Figure 2D).

Subsequently, KEGG database pathways and GO enrichment analysis were performed based on the differential genes. As depicted in Figure 2E, the top 10 GO enrichment analysis encompassed developmental growth involved in morphogenesis, cell cycle checkpoint signaling, regulation of binding, nucleocytoplasmic transport, nuclear transport proteasome‐mediated ubiquitin−dependent protein, extracellular exosome biogenesis, regulation of intracellular, protein transport regulation of neuron projection development, regulation of cell size, and positive regulation of DNA metabolic process. The top 10 KEGG pathways (Figure 2F) included Pathways of neurodegeneration—multiple diseases, MAPK signaling pathway, Endocytosis, Exosome mediated excretion, Focal adhesion, Pathogenic *Escherichia coli* infection, Axon guidance, Autophagy–animal, Cell cycle, and Apoptosis.

To investigate the potential growth‐inducing effects of exosomes on HCC, exosomes were isolated from various cultured media using an exosome isolation kit and subsequently introduced into the medium used in this study. A clone formation analysis was conducted, revealing that the presence of lupeol (40 μM) significantly inhibited the proliferation of HepG2 cells (*p* < 0.05). However, in the lupeol + exosome group, it was observed that exosomes counteracted the inhibitory effects of lupeol and instead promoted the proliferation of HepG2 cells. Therefore, our subsequent investigation aims to elucidate the mechanistic interactions between lupeol and exosomes in HCC (Figure 2G).

### 3.3 Lupeol Causes Cells Death by Inhibiting Exosomes Formation

**FIGURE 3:**
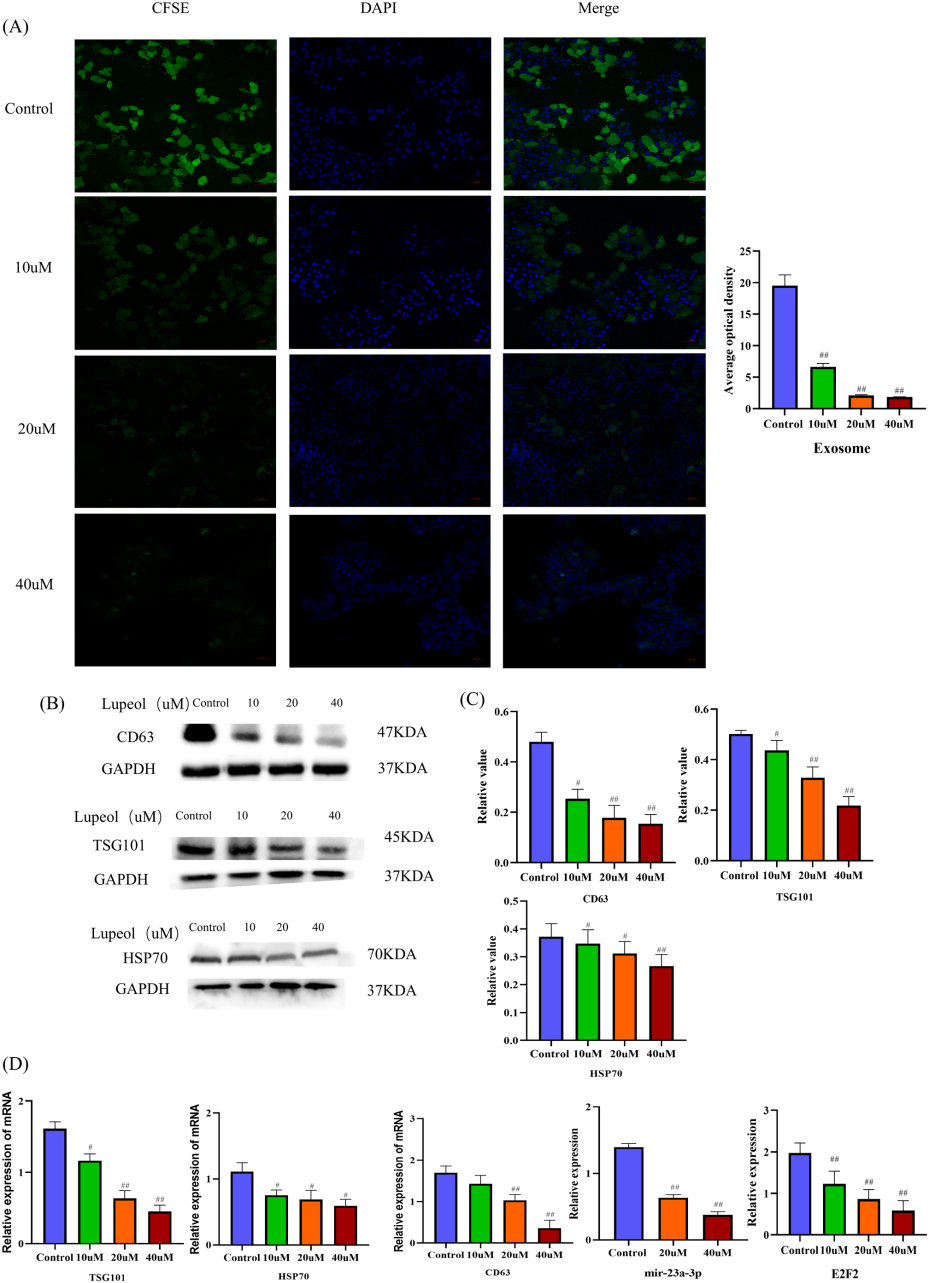
Lupeol has been found to exert inhibitory effects on the proliferation of HepG2 cells through the inhibition of exosome secretion. (A) Immunofluorescent findings of exosome flux (B, C) WB analysis of exosome‐related proteins CD63, TSG101, and HSP70 in HepG2 cells treated with varying concentrations of lupeol. The obtained results were analyzed using prism 8.0.2, with statistical significance indicated by ^#^ *p* < 0.05 and ^##^ *p* < 0.01, when compared to the control group. (D) RT‐PCR analysis of the mRNA expression levels of CD63, TSG101, HSP70, mir23a‐3p, and E2F2 were assessed, with statistical significance indicated by ^#^ *p* < 0.05 and ^##^ *p* < 0.01, compared to the control group.

Given the close relationship of exosomes and HCC, we sought to investigate the mechanism behind proliferation and migration inhibition caused by lupeol in HepG2 cells. Firstly, the immunofluorescence of exosomes was detected using confocal laser scanning microscopy, while the findings indicate a significant decrease in the average optical density of exosomes as the concentration of lupeol increases (*p* < 0.05) (Figure 3A). To validate the identity of the isolated vesicles, we analyzed three distinct exosome markers recommended by EV guidelines: the transmembrane protein CD63, the cytosolic biogenesis marker TSG101, and the stress protein HSP70. As shown in Figure 3B,C, Western blot analysis confirmed the presence of these markers in the isolated fractions. Upon treatment with increasing concentrations of Lupeol, we observed a significant, dose‐dependent decrease in the expression of all three markers (CD63, HSP70, and TSG101) (*p* < 0.05). This consistent downregulation across multiple specific markers strongly indicates that Lupeol inhibits the secretion of bona fide exosomes. Additionally, the mRNA expression levels of CD63, HSP70, TSG101, mir‐23a‐3p and E2F2 were observed to be down‐regulated with increasing concentrations of lupeol (*p* < 0.05) (Figure 3D). It is well‐established that CD63, TSG101, and HSP70 are enriched in exosomes, and their increased expression serves as a specific marker for exosome formation (Doyle and Wang 2019). Also, mir‐23a‐3p serves as a major constituent within exosomes, facilitating migration in HCC, and the down‐regulation of mir‐23a‐3p results in the inhibition of exosome function (Dai et al. 2020). E2F2, a member of the E2F2 transcription factor family (Liu et al. 2022), has been investigated to show that restraining the expression of E2F2 improves autophagic formation.

### 3.4 Lupeol Inhibited the Exosomes Through Inducing Autophagic

**FIGURE 4:**
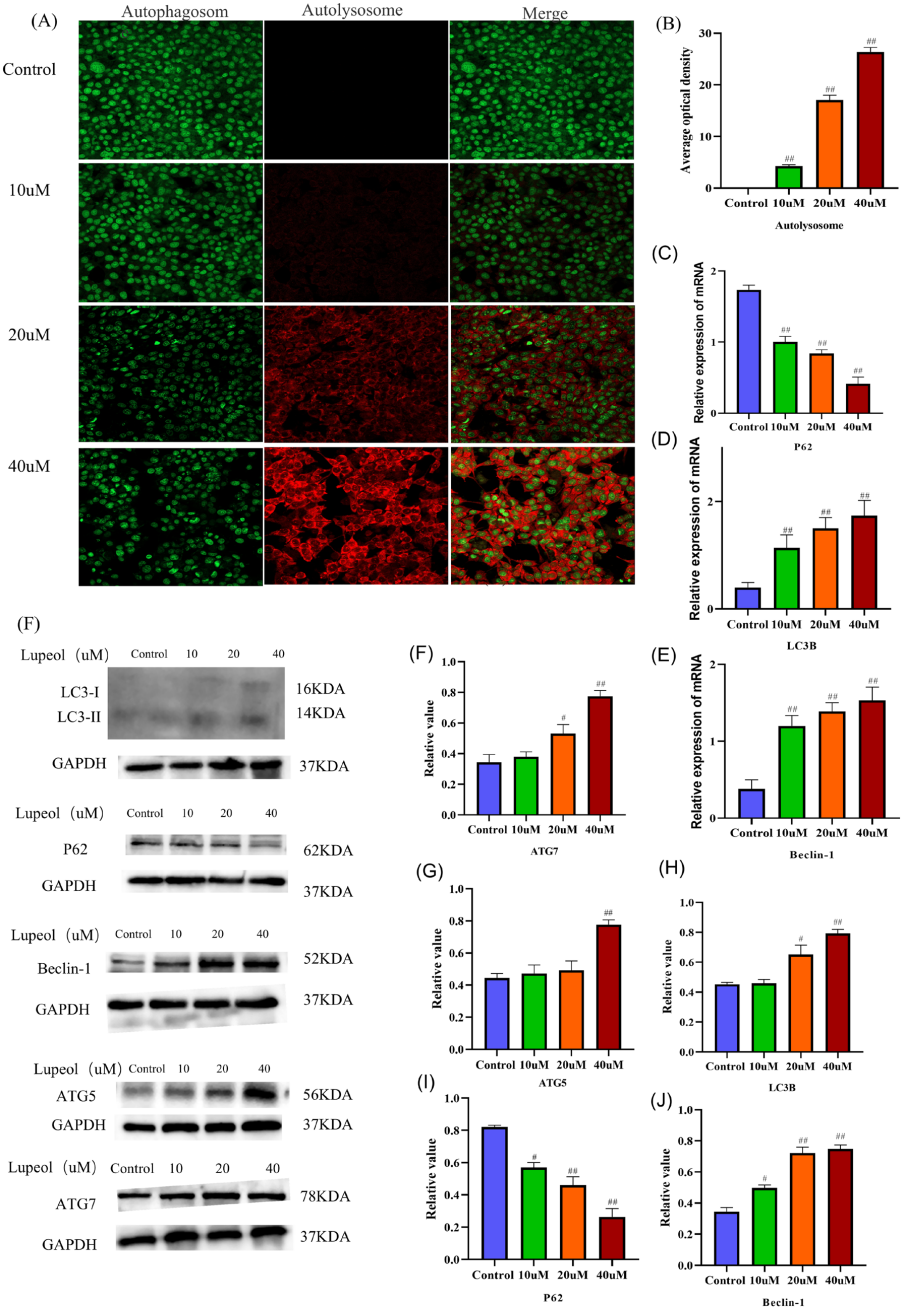
Lupeol inhibits HepG2 cell proliferation by inducing autophagy. (A) Immunofluorescent findings of autophagic flux; (B) WB analysis of level of autophagy‐related proteins LC3B, P62, Beclin‐1, ATG7 and ATG5 in HepG2 cells treated with different concentrations of lupeol, with the results analyzed by Prism 8.0.2, ^#^ *p* < 0.05, ^##^ *p* < 0.01, compared with the control group, (D–H) the expression mRNA LC3B, P62 and Beclin‐1, ^#^ *p* < 0.05, ^##^ *p* < 0.01, compared with the control group. (C) RT‐PCR analysis of the mRNA of LC3B, P62, and Beclin‐1 was assessed, with the results analyzed by Prism 8.0.2, ^#^ *p* < 0.05, *^##^p* < 0.01, compared with the control group.

To further investigate the underlying cause, we conducted an examination of the autophagic flow. Subsequently, shown in Figure 4A–B, the immunofluorescence results revealed a significant increase in the average optical density of autolysosomes as the concentrations of lupeol increased (*p* < 0.05). As shown in Figure 4F–J, the western‐blot results indicate that the expressions of LC3B, Beclin‐1, ATG5, and ATG7 were significantly increased with increasing concentrations of lupeol (*p* < 0.05). It is widely recognized that LC3 plays a crucial role in the formation of autophagy. Increasing the ratio of LC3II/LC3I has been shown to elevate the level of autophagy (Schaaf et al. 2016). Additionally, Beclin‐1 has been identified as a key initiator of autophagy; enhancing the expression of Beclin‐1 has been found to promote autophagic formation (Levine et al. 2015). Conversely, the expression of P62 was significantly decreased with increasing concentrations of lupeol (*p* < 0.05). The enhanced autophagic formation leads to a decrease in P62, which serves as a substrate of autophagy (Lamark et al. 2017). Moreover, in the Figure 4C–E, the mRNA levels of LC3B and Beclin‐1 were up‐regulated with increasing concentrations of lupeol (*p* < 0.05), while the mRNA levels of P62 were down‐regulated with increasing concentrations of lupeol (*p* < 0.05).

**FIGURE 5:**
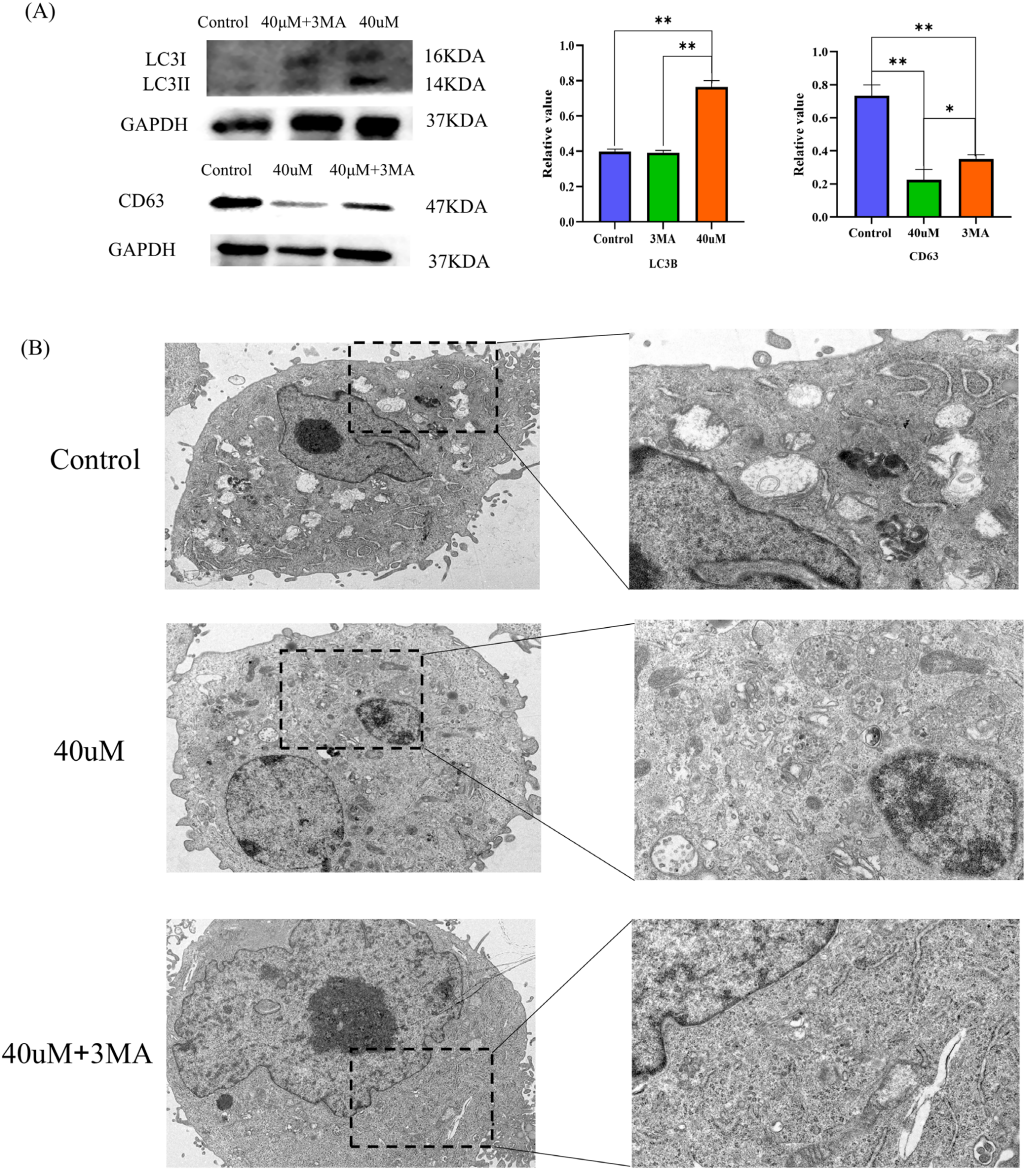
Lupeol induced autophagy to restrain the expression of exosome gene. (A) WB analysis of the level of autophagy‐related protein LC3B and exosome‐related protein CD63 in HepG2 cells treated with different concentrations of lupeol with the results analyzed by prism 8.0.2, ^*^ *p* < 0.05, ^**^ *p* < 0.01, compared with the control group. (B) The results of transmission electron microscope.

To confirm the causal link between autophagy induction and exosome suppression, we employed the autophagy inhibitor 3‐MA to block the autophagic flux. As shown in Figure 5A, Western blot analysis revealed that Lupeol treatment (40 μM) significantly upregulated LC3B and downregulated the exosome marker CD63. However, co‐treatment with 3‐MA effectively reversed these effects, leading to a restoration of CD63 expression levels compared to the Lupeol‐only group (*p* < 0.05). This rescue effect suggests that the suppression of exosome secretion by Lupeol is dependent on the activation of autophagy. Consistently, the introduction of 3‐MA also partially reversed the inhibitory effects of Lupeol on colony formation and cell migration (*p* < 0.05). To further characterize the vesicles, transmission electron microscopy (TEM) was performed. As shown in Figure 5B, the control group exhibited vesicles with a typical cup‐shaped morphology and double‐membrane structure, characteristic of exosomes. In the Lupeol‐treated group (40 μM), the density of these exosome‐like vesicles was markedly reduced, while the number of intracellular autophagosomes increased. Importantly, co‐treatment with the autophagy inhibitor 3‐MA restored the presence of these cup‐shaped vesicles in the extracellular space, visually confirming the Western blot results. These results collectively demonstrate that Lupeol suppresses exosome secretion specifically through the induction of autophagy.

### 3.5 Lupeol Suppresses Tumor Growth of HepG2 Cells In Vivo

**FIGURE 6:**
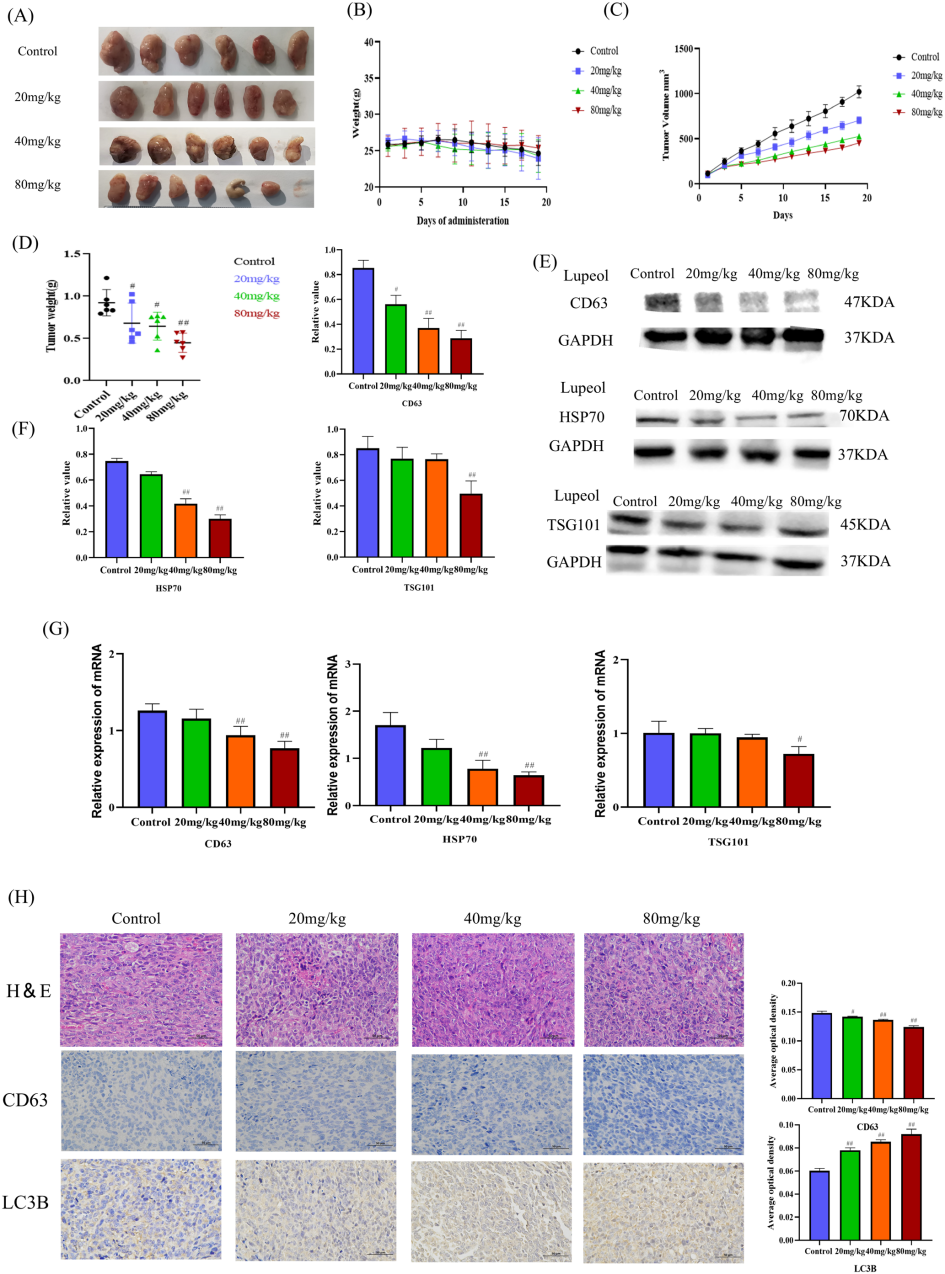
Lupeol inhibits HepG2 proliferation in vivo. (A, B) weight growth changes of mice; (C) Lupeol inhibits tumor growth in subcutaneous xenograft models and xenograft tumor volumes were measured every 2 days; (D) weight of tumors. (E, F) WB analysis of level of autophagy‐related proteins of CD63, TSG101, and HSP70 in HepG2 cells treated with different concentrations of lupeol, with the results analyzed by prism 8.0.2, ^#^ *p* < 0.05, ^##^ *p* < 0.01, compared with the control group, (G) RT‐PCR analysis of the mRNA expression levels of CD63, TSG101 and HSP70 were assessed, with statistical significance indicated by ^#^ *p* < 0.05 and ^##^ *p* < 0.01, compared to the control group. (H) H&E staining results of tumors (400×).

**FIGURE 7:**
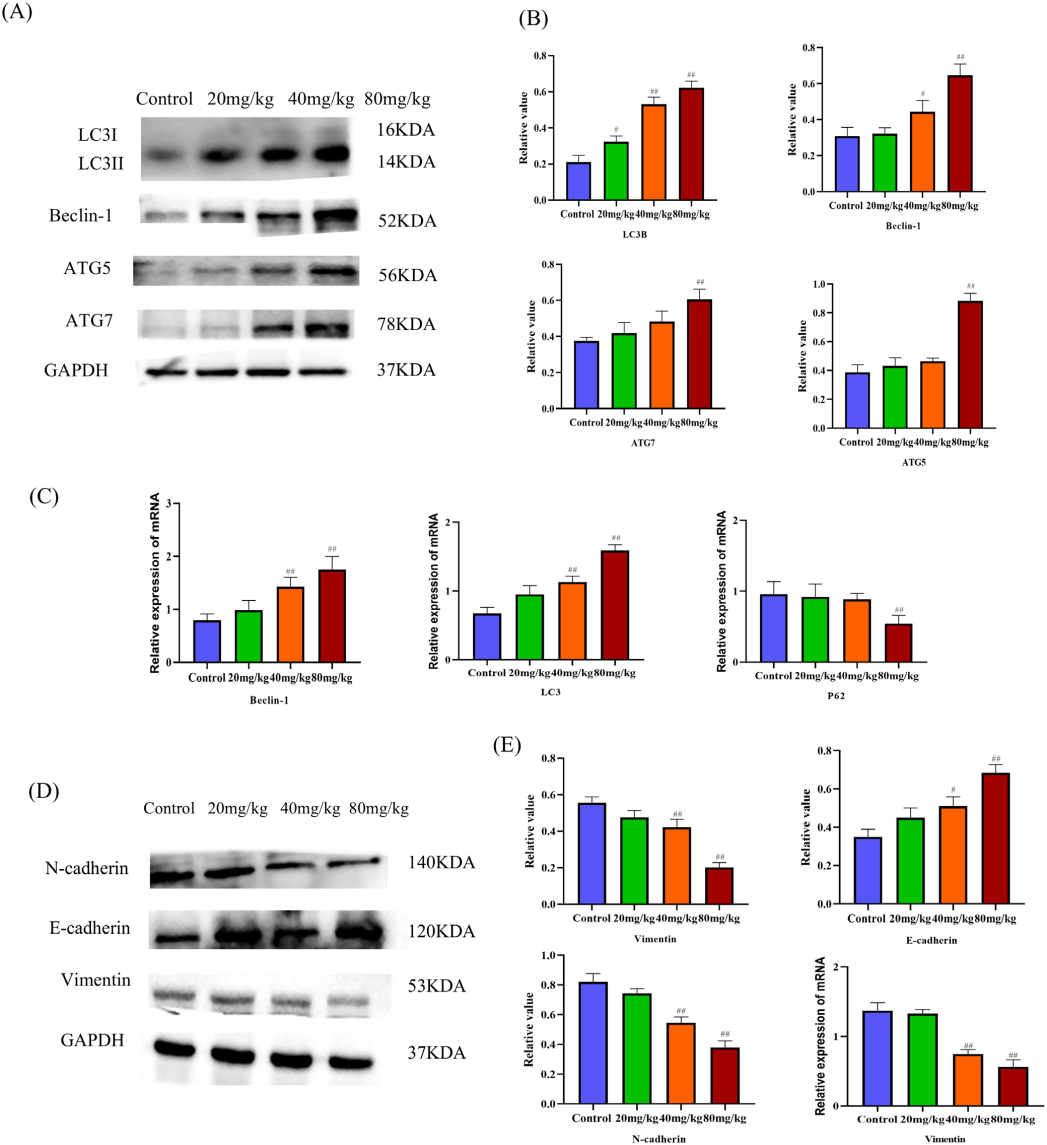
(A, B) WB analysis of the levels of the autophagy‐related proteins LC3B, P62, Beclin‐1, ATG7 and ATG5 in tumors treated with different concentrations of lupeol, with the results analyzed by Prism 8.0.2, ^#^ *p* < 0.05, ^##^ *p* < 0.01, compared with the control group. (C) RT‐PCR analysis of the mRNA expression levels of LC3B, P62 and Beclin‐1, ^#^ *p* < 0.05, ^##^ *p* < 0.01, compared with the control group. (D, E) WB analysis of the levels of the EMT‐related proteins N‐cadherin, E‐cadherin and Vimentin in tumors treated with different concentrations of lupeol, with the results analyzed by Prism 8.0.2, ^#^ *p* < 0.05, ^##^ *p* < 0.01, compared with the control group.

To assess the inhibitory effects of lupeol on the growth of HepG2 in vivo, a study was conducted using HepG2 tumor‐bearing mice. The mice were divided into different treatment groups, with lupeol administered at doses of 20, 40, and 80 mg/kg, while the control group received corn oil. The results, as depicted in Figure 6A,C,D, demonstrated that treatment with lupeol significantly suppressed the growth and weights of HepG2 tumors in a dose‐dependent manner, when compared to the control group (*p* < 0.05). Notably, there were no significant differences observed in the bodyweight of the mice across the various treatment groups (Figure 6B). The findings from the Immunohistochemistry analysis revealed a significant up‐regulation in the expression of LC3B and a down‐regulation in the expression of CD63, as the concentration of lupeol increased in comparison to the control group (*p* < 0.05), shown in Figure 6H. According to the results obtained from the western blot analysis and Real‐time PCR (Figure 6E–G), the inhibition of exosome expression was found to result in the down‐regulation of CD63, HSP70, and TSG101 at various concentrations of lupeol treatment (*p* < 0.05). Additionally, the levels of autophagy proteins were observed to be promoted, as evidenced by the up‐regulation of LC3B, Beclin‐1, ATG5, and ATG7, and the down‐regulation of P62 with different concentrations of lupeol treatment (*p* < 0.05) Figure 7A,B. Furthermore, the inhibition of migration was associated with the up‐regulation of E‐cadherin and the down‐regulation of N‐cadherin and Vimentin upon exposure to lupeol treatment (*p* < 0.05), as shown in Figure 7D,E. The findings from the analysis of mRNA expression levels of LC3B, Beclin‐1 were up‐regulated with different concentrations of lupeol treatment (*p* < 0.05), the mRNA expression levels of P62, CD63, HSP70, TSG101, and Vimentin were down‐regulated with different concentrations of lupeol treatment (*p* < 0.05), These results indicate a significant correlation with the western blot results, as shown in Figures 6G and 7C.

### 3.6 Convergence of Gene Expression Signatures Across Different Studies of HCC

**FIGURE 8:**
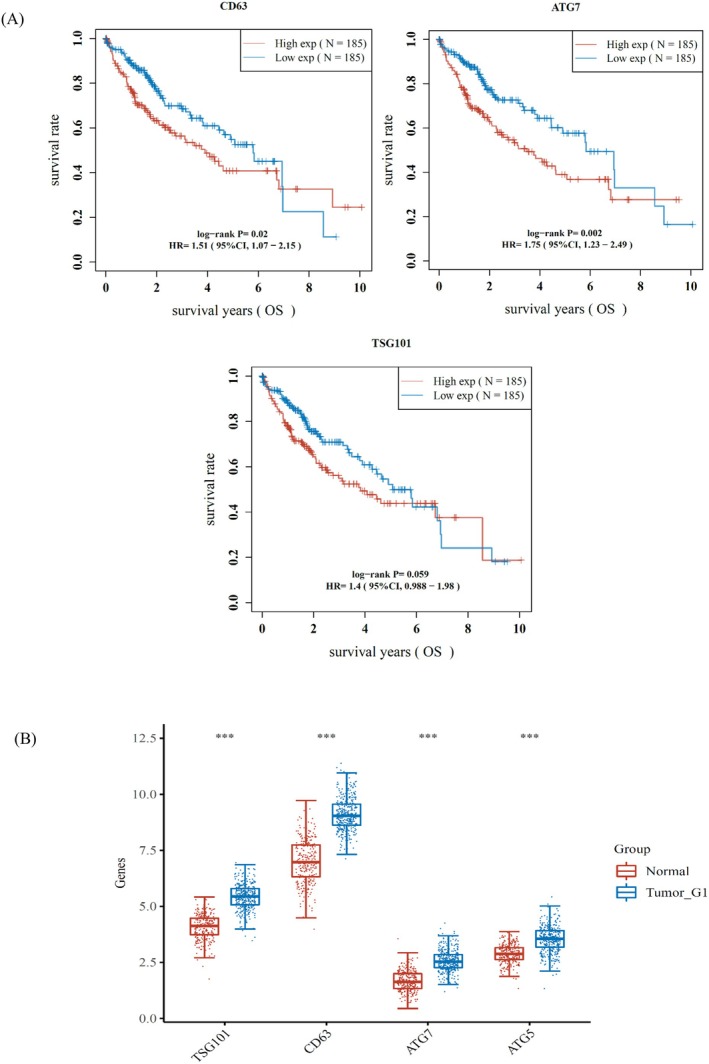
The expression of genes and survival analysis in TCGA data. The data distribution of standardized TPM data is close to normal distribution. (A) The results of the Boxplot show that the expression distribution of CD63, TSG101, ATG5 and ATG7 genes in tumor tissues and normal tissues. The abscissa represents different groups of samples, and the ordinate represents the expression distribution of genes; different colors represent different groups. ***p < 0.001, asterisks (*) stand for significance levels. The statistical difference of two groups was compared through the Wilcox test. (B) Kaplan–Meier survival analysis of the CD63, TSG101 and ATG7 gene signature from the TCGA dataset.

All RNAseq data and clinical information were acquired from the TCGA database. As shown in Figure 8A, the tumor group exhibited significantly higher expression of the CD63, TSG101, ATG5, and ATG7 genes compared to the normal group (*p* < 0.001). Additionally, the results of the survival analysis are shown in Figure 8B, indicating that the exosome‐related gene CD63 (*p* < 0.05) and autophagy‐related gene ATG7 (*p* < 0.05) have high prognostic value. However, the exosome‐related gene TSG101 (*p* > 0.05) does not have a significant prognostic value.

## 4 Discussion

HCC is a prevalent malignancy among different types of cancers (Vogel et al. 2022). In clinical practice, several strategies have been employed for the diagnosis and treatment of HCC, such as immune checkpoint inhibitors (ICIs) (Harkus et al. 2022), which are designed to counteract tumor immune evasion. However, the overall response rate of advanced HCC to ICIs remains relatively low. Given the lack of specific molecular targets and the high mortality rate associated with HCC (Kumar et al. 2025), treating this condition poses a significant challenge. Consequently, there is a pressing need to explore innovative therapeutic strategies to address this issue. Notably, lupeol has shown promise in inhibiting tumor functions, suggesting its potential utility in restraining HCC. Therefore, investigating the mechanisms underlying the treatment of HCC becomes imperative.

Previous research has indicated that exosomes serve as cell communication messengers and may play a significant role in promoting tumor growth, particularly in HCC (Sasaki et al. 2019). Our results showed that exosomes derived from tumor cells enhanced the migration and proliferation of HepG2 cells, while the effects of exosomes were attenuated by the presence of lupeol. Exosomes consist of various components such as proteins, RNA, microRNA, and others. Among these, CD63, TSG101, and HSP70 proteins serve as markers for exosomes, indicating their presence in tumor cells. Additionally, the release of mir‐23a‐3p from exosomes has been linked to tumor growth, as demonstrated by previous studies (Sun et al. 2018). However, the underlying mechanisms responsible for this phenomenon remain unknown, necessitating further investigation to elucidate the causative factors. Recent studies have highlighted a competitive relationship between autophagy and exosome biogenesis, as both processes rely on the formation and trafficking of MVBs (Buratta et al. 2020). Under normal conditions, MVBs can fuse with the plasma membrane to release exosomes. However, when autophagy is induced, MVBs are increasingly targeted to lysosomes for degradation (secretory autophagy), thereby reducing exosome release. Our study provides mechanistic evidence supporting this “switch” in HCC cells. We observed that Lupeol treatment significantly upregulated autophagy markers (LC3‐II, Beclin‐1) while downregulating exosome markers (CD63, TSG101). Importantly, our inhibitor experiments support a mechanistic link between autophagy activation and exosome suppression. We observed that blocking autophagy with 3‐MA significantly attenuated the Lupeol‐induced reduction of CD63 and restored exosome secretion (Figure 5). This suggests that Lupeol likely functions by redirecting MVBs toward the autophagic‐lysosomal pathway, thereby limiting the release of exosomes that would otherwise support cell proliferation and migration.

The intricate relationship between autophagy and exosome production has been extensively studied, with a growing body of evidence indicating that these two processes are closely interconnected and can significantly influence human health (Salimi et al. 2020; Xing et al. 2021). Exosomes, which are extracellular vesicles primarily found in bodily fluids such as blood, play a crucial role in various biological processes, including immune regulation, antigen presentation, and cell migration. Autophagy, on the other hand, is an intracellular degradation process that promotes cell survival by degrading soluble proteins and other organelles under various stress signals (Wu et al. 2021). The crosstalk between exosomes and autophagy is highly complex and depends primarily on the cellular environment. This relationship is bidirectional, meaning that exosomes can regulate the intracellular autophagic process, and vice versa (Wang et al. 2019). However, recent research has focused on how autophagy can inhibit the production of exosomes. One of the key mechanisms by which autophagy inhibits exosome production is through the regulation of the endosomal sorting complex required for transport (ESCRT) pathway. The ESCRT pathway is responsible for the formation and secretion of MVBs, which are essential for the formation of exosomes (Gondaliya et al. 2023). Autophagy can regulate the ESCRT pathway by affecting the trafficking and sorting of proteins within the endosomal system. This can lead to a decrease in the formation of MVBs and subsequently a reduction in exosome production (Ghebosu et al. 2024). Additionally, autophagy can also inhibit exosome production by affecting the availability of membrane sources for exosome biogenesis. Exosomes are formed by the inward budding of the plasma membrane into the cytoplasm, which is then pinched off to form the exosomal vesicle. Autophagy can regulate the availability of membrane sources by affecting the recycling of endosomal membranes back to the plasma membrane (Jahangiri et al. 2022).

In our study, we have demonstrated that lupeol induces autophagy to suppress the secretion of tumor cell‐derived exosomes in HCC. However, emerging research suggests that tumor cell‐derived exosomes play a role in remodeling the tumor microenvironment, such as activating the STAT3 pathway through high levels of IL‐6‐exosomes, leading to M2 polarization of macrophages and promoting cancer progression (He et al. 2022). Additionally, these exosomes induce angiogenesis through stimulation of the PI3K/Akt axis, thereby facilitating tumor progression (Zhang, Chen, et al. 2022). Therefore, our objective of the subsequent phase entails an examination of the mechanisms through which lupeol interacts with the tumor microenvironment, with the aim of further elucidating the potential of lupeol. In breast cancer, exosomes can stimulate autophagy to facilitate tumor invasion via the activation of the AMPK/mTOR pathway by miR‐126 (Fu and Tong 2020). Additionally, in a hypoxic environment, upregulating the expression of exosomal miR‐30a can decrease apoptosis in myocardial cells (Wang et al. 2020). Exosomes derived from tumors have been found to facilitate tumor proliferation and migration primarily through the activation of cancer‐promoting ligands, which in turn activate target cells, or through the secretion of miRNA that alters the gene grid configuration (Whiteside 2021; Zhou et al. 2020). Current research increasingly indicates that the miRNA secreted by tumor‐derived exosomes is closely associated with cancer EMT‐related pathways (Yang et al. 2021).

Our findings suggest that Lupeol‐induced autophagy acts as a critical upstream regulator that suppresses exosome secretion, which subsequently contributes to the inhibition of HCC cell proliferation and migration. While this study provides robust evidence linking autophagy to exosome suppression using pharmacological inhibitors and multiple protein markers (CD63, TSG101, HSP70), we acknowledge certain limitations. Specifically, Nanoparticle Tracking Analysis was not performed to quantify the particle size distribution and absolute concentration of exosomes. Future studies utilizing NTA and in vivo exosome tracking will further strengthen the biophysical profiling of Lupeol‐regulated vesicles.

In conclusion, the collective findings presented herein indicate that Lupeol impedes the proliferation of HCC cells, at least in part, by inducing autophagy to restrain exosome secretion. Additionally, Lupeol has been shown to inhibit HCC metastasis by reducing the expression of EMT. This investigation has unveiled the innovative potential of Lupeol as an effective therapeutic agent for HCC, as it effectively curtails exosome secretion via autophagy induction. Consequently, these findings offer valuable insights into the treatment and prognosis of HCC.

## Author Contributions

J.‐S.W. and L.‐L.W.: all the authors have made contributions to the manuscript and approved the version to be submitted. J.‐S.W. and L.‐L.W.: the co‐corresponding authors, responsible for designing the experiments and providing the funding support. X.Z. and K.C.: the co‐first author, responsible for writing the article and preparing figures. C.Z, D.‐y.L.: responsible for analyzing the data.

## Funding

This work was supported by the Guangdong Basic and Applied Basic Research Foundation (2023A1515110307 and 2024A1515140046).

## Conflicts of Interest

The authors declare no conflicts of interest.

## Data Availability

The datasets generated during and/or analyzed during the current study are available from the corresponding author on reasonable request.
